# Ontogenesis from Embryo to Juvenile in Threadsail Filefish, *Stephanolepis cirrhifer*

**DOI:** 10.3390/ani15081124

**Published:** 2025-04-13

**Authors:** Liming Liu, Xuanhan Liu, Yanqing Wu, Jun Zeng, Wengang Xu

**Affiliations:** 1 School of Ocean, Yantai University, Yantai 264005, China; liu_liming71@163.com (L.L.); 15898031011@163.com (X.L.); 2 Yantai Engineering Research Center of Deep-Sea Aquaculture of Economic Fish, Yantai 264005, China; 3 Shandong Engineering Research Center of Healthy Land-Sea Relay Farming of Economic Fish, Yantai 264005, China; 4 East China Sea Fisheries Research Institute, Chinese Academy of Fishery Sciences, Shanghai 200090, China; wuyanqing0961@163.com; 5 Guangxi Academy of Marine Sciences, Guangxi Academy of Sciences, Nanning 530007, China; junzeng@gxas.cn; 6 Beibu Gulf Marine Industrial Research Institute, Fangchenggang 538000, China

**Keywords:** growth, morphology, hatching, larvae, juvenile, filefish

## Abstract

The threadsail filefish, *Stephanolepis cirrhifer*, is an economically important marine species. However, wild catches have sharply decreased over the past 20 years, causing *S. cirrhifer* to be added to the IUCN Red List of Threatened Species. Accordingly, this study seeks to promote technological development for artificial breeding and early life-stage farming by defining the morphological characteristics of ontogenesis. The fertilized eggs, with a diameter of 0.62 ± 0.01 mm, were spherical and sticky and contained multiple oil globules of varying sizes. The embryonic development was observed and divided into eight phases, which were cleavage, blastocyst, gastrula, neurula, organogenesis, muscular contraction, heart pulsation, and hatching. At 3 days post-hatching (dph), the yolk sac was completely absorbed. The eye developed rapidly, and the mouth fissure and anus initially formed. Some larvae were fed on S-rotifers (*Brachionus plicatilis*). At 6–8 dph, the upper and lower jaws of larvae were gradually covered by leathery skin, and the head-to-body proportion increased. At 14–16 dph, the fin differentiation occurred in the dorsal, anal, and pectoral fins, with widespread distribution of yellow and melanin on the body surface. Swim bladder was clear. The swimming ability of larva was enhanced, resulting in an obvious clustering phenomenon. At 22–25 dph, the end of the notochord continued to tilt upwards, forming a tail fin. The trunk was evenly distributed with protruding circular punctate scales. The snout was covered with leathery epidermis, and the mouth began to round. At 40–45 dph, the juvenile completed metamorphosis, with horizontal dark stripes appearing on the trunk. Pigmented spots appeared on the tail fins. The counts of dorsal and anal fin spines were 34–36 and 32–34 dph, respectively. During the development of larvae and juveniles, the growth parameters, such as total length, standard length, body height, and body weight, were made as growth curves. The slopes of growth curves were calculated. We found two inflexion points occurring in the growth curves, which may be associated with metamorphosis and transitions in feeding habits. These results enrich the biological understanding of filefish species while providing guidance for artificial propagation and fry production in *S. cirrhifer*.

## Simple Summary

This study details and evaluates the morphological characteristics of the early embryonic development and ontogenesis of *Stephanolepis cirrhifer*, commonly known as the threadsail filefish. This species has experienced a considerable reduction in numbers in recent decades to the point of being listed as a threatened species in 2017, yet techniques for developing artificial propagation of the species have remained elusive. Our findings probably define the morphological characteristics of ontogenesis from embryo to juvenile. The embryonic development was observed and divided into eight phases, which were cleavage, blastocyst, gastrula, neurula, organogenesis, muscular contraction, heart pulsation, and hatching. Additionally, we found that the weaning period occurred from 40 dph, and two inflexion points occurring in the growth curves of larvae and juveniles were found to be associated with metamorphosis and transitions in feeding habits.

## 1. Introduction

Filefish belong to the Monacanthidae family, comprising approximately 102 species and 32 genera distributed worldwide, with most species inhabiting coastal reefs, coral reefs, and sand sea areas [1]. In particular, the threadsail filefish, *Stephanolepis cirrhifer* (Temminck and Schlegel, 1850), is mainly distributed in the Western Pacific waters from Hokkaido to the East China Sea [2,3,4] and is an important commercial species in Asian countries, especially in Japan and South Korea [5,6,7,8]. In Japan, the liver and sashimi of *S. cirrhifer* are commonly used as premium ingredients [9], whereas in South Korea, dried *S. cirrhifer* fish products are popular [10]. Furthermore, *S. cirrhifer* prey on jellyfish larvae, with the potential to inhibit jellyfish booms [4,11].

Owing to overfishing, habitat destruction, and marine environmental pollution, wild catches of *S. cirrhifer* have sharply decreased from a collective output in excess of 250,000 tons in the Northwest Pacific in 1985 to below 350 tons in 2002 [12]. Similarly, in 2009, the annual *S. cirrhifer* catch dropped to 1500 tons, representing a 99% decrease from 1990 to 2009 in South Korea [13] and a corresponding 99% decrease in wild catch output. Consequently, in 2017, *S. cirrhifer* was placed on the IUCN Red List of Threatened Species [14].

Over the past decade, studies on *S. cirrhifer* have focused on its external morphology [15,16], genetic characteristics, population genetic structure [17,18], acoustic target strength [19], and feed nutrient levels [20,21]. This has led to the development of artificial culture and breeding techniques to increase production and maintain resource balance [22,23]. For example, one study reported that artificially cultured *S. cirrhifer* grew rapidly, reaching the commercial standard within 1 year, with a higher-quality liver than wild species [8]. In addition, *S. cirrhifer* exhibit cage-cleaning properties by feeding on attached algae and small animals. Hence, improved *S. cirrhifer* farming has important implications for human consumption and cage-cleaning processes. However, given that the artificial breeding technology has not yet achieved complete success, the main source of fish larvae depends on wild catches [18], and large-scale cultivation of *S. cirrhifer* remains to be realized.

Our previous study considered the changes in the growth and feeding characteristics during early ontogenesis in *S. cirrhifer* [24]. To improve the survival rate of larvae, the optimization of the processes of early embryonic development and ontogenesis after hatching is critical to fish culture. To date, few studies have provided a detailed characterization of these life stages for *S. cirrhifer*. Meanwhile, related processes have been thoroughly investigated in rockfish [25] and flatfish [26], both of which have been successfully cultivated on a large scale. Therefore, studies on the early development of fish species are needed to understand their basic biology, feeding requirements, and environmental preferences to develop successful artificial breeding strategies [27,28]. Accordingly, herein, we describe the morphological development and growth of *S. cirrhifer* throughout the early life stages.

## 2. Materials and Methods

### 2.1. Acquisition of Fertilized Eggs

The present study was conducted at Yantai Laizhou Aquatic Co., Ltd. in Shandong, China, in 2020. One hundred female and one hundred male wild parental *S. cirrhifer* caught in the Yellow Sea were selected for indoor ripening cultivation in November 2019. The average body weights of female and male fish were 520.17 ± 56.30 g and 401.15 ± 46.19 g, respectively. The fish were fed with fresh mussel *Mytilus edulis* and oyster *Ostreidae* daily, together with live sandworms *Nereis succinea* to promote gonadal maturation. Male and female fish were naturally mated and spawned after 3 months in February 2020. Polyethylene sheets were placed at the bottom of the pond and used as attachment bases for the collection of the fertilized eggs. Once several eggs were attached to the sheets, they were immediately removed and placed in an incubator.

### 2.2. Egg Incubation

The fertilized eggs were incubated in a 20 m^3^ concrete pool with gentle aeration and circulating seawater. The seawater was purified through two sand filters (DS-1200, Yixing Bohui Environmental Protection Technology Co., Ltd., Yixing, China) connected in series, and the salinity was 33 ± 0.5 psu. The water temperature and pH were maintained at 23.6 ± 0.5 °C and 7.8–8.2, respectively. The light intensity at the water surface was maintained at 5000–8000 lx using fluorescent lamps (E27, Opple Co., Ltd., Zhongshan, China). Furthermore, a flow rate of 40–50% seawater exchange/day was applied in this study.

### 2.3. Culture of Larvae and Juveniles

From hatching to 3 days post-hatching (dph), the larvae mostly rely on their own yolk sac as nutrition for survival. From 3 dph, they started to open their mouths for feeding. Based on the larval size, they were fed the rotifer (*Brachionus plicatilis*) at a density of 1–2 ind/mL in water from 3 to 25 dph, *Artemia salina* nauplii at a density of 0.5–1 ind/mL from 25 to 40 dph, and formula feed after 40 dph. During the changes in the feeding regimes, the proportion of two types of food was half each.

The cultivation condition was shaded, and 20 W LED lamps were used to adjust light intensity. A portable water quality detector (Hengxin, 86031, AZ Instrument Corp., Taiwan, China) was used daily to measure water temperature, salinity, dissolved oxygen, and pH. The water quality parameters were as follows: temperature, 24.1–25.2 °C; salinity, 31.4–32.2 psu; pH, 8.00–8.27; dissolved oxygen levels, 5.0–5.9 mg/L. The aeration was maintained for 24 h, and seawater exchange rate was gradually increased from 10% to 150%/d at the settling stage of juveniles.

### 2.4. Observation of Embryonic Development and Larval/Juvenile Morphology

At each developmental stage of embryos and larvae, 20 specimens were sampled daily from the incubation and larval rearing cement pool, respectively. The morphological characteristics and duration of development stages of larvae and juveniles were observed and photographed using a light microscope (Nikon SMZ800, Nikon, Tokyo, Japan). A digital CCD camera (Nikon US300) and VImage 2014 software (Vezu Technology Co., Ltd., Guangzhou, China) was used to capture images and measure the diameters of the oil globules and yolk sacs. The growth indices of each sampled larva, such as total length (TL), standard length (SL), body height (BH), were measured. The body weight (BW) of larva was measured using an electronic balance (ME55; Mettler-Toledo International Inc., Zurich, Switzerland) with a sensitivity of 0.01 mg.

### 2.5. Image Processing and Statistical Analysis

The larvae were placed in a culture dish, and photographs of the developmental stages were implemented using Photoshop CS6 (Adobe Systems, San Jose, CA, USA). All data were analyzed using SPSS v26.0 software (IBM, Armonk, NY, USA). Data are expressed as the mean ± standard deviation (SD). The TL, SL, BH, and BW were plotted against age. Linear and nonlinear regressions were performed using OriginPro 9.1 (OriginLab Corp., Northampton, MA, USA). The inflexion points were calculated according to Van Snik et al. [29].

## 3. Results

### 3.1. Embryonic Development

**Figure 1:**
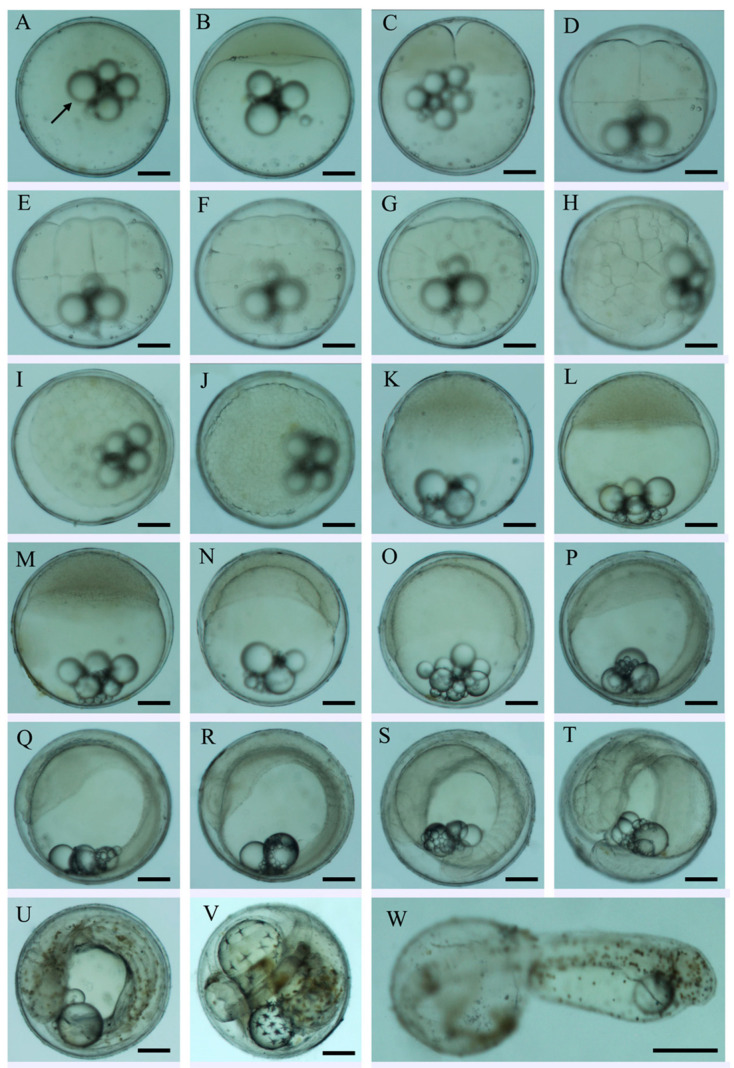
Embryonic development of *S. cirrhifer*. (**A**) Fertilized egg; (**B**) blastodisc formation; (**C**) 2-cell; (**D**) 4-cell; (**E**) 8-cell; (**F**) 16-cell; (**G**) 32-cell; (**H**) 64-cell; (**I**) 128-cell; (**J**) morula stage; (**K**) high blastula; (**L**) low blastula; (**M**) early gastrula; (**N**) middle gastrula; (**O**) late gastrula; (**P**) blastopore closed; (**Q**) formation of somite; (**R**) formation of optic vesicle; (**S**) tail-bud appearing; (**T**) formation of eye lens; (**U**) muscular contraction; (**V**) heart pulsation; (**W**) hatching. The arrow marked in (**A**) shows the appearance of oil globules. Scale bars = 0.2 mm.

The total 120,000 fertilized eggs of *S. cirrhifer* were spherical, sticky, and demersal, and the diameter of eggs was 0.62 ± 0.01 mm (Figure 1A). The yolk was transparent and contained multiple oil globules of varying sizes. The ooplasm was evenly distributed, and the membrane was thick, comprising 4–6 oil globules (100–142 μm) and 8–12 smaller oil globules (<20 μm). Unfortunately, we failed to count the survival rate of embryos at each developmental stage from fertilized egg to hatching in this study.

At 23.6 °C, it took about 48 h for the eggs to develop into larvae. The embryonic characteristics and duration of development in each stage of *S. cirrhifer* are shown in Figure 1 and Table 1, referred to from our recent study focusing on the species of Senegalese sole *Solea senegalensis* [30]. The hatching rate was about 70–85%.

### 3.2. Morphological Characteristics of Larvae and Juveniles

**Figure 2:**
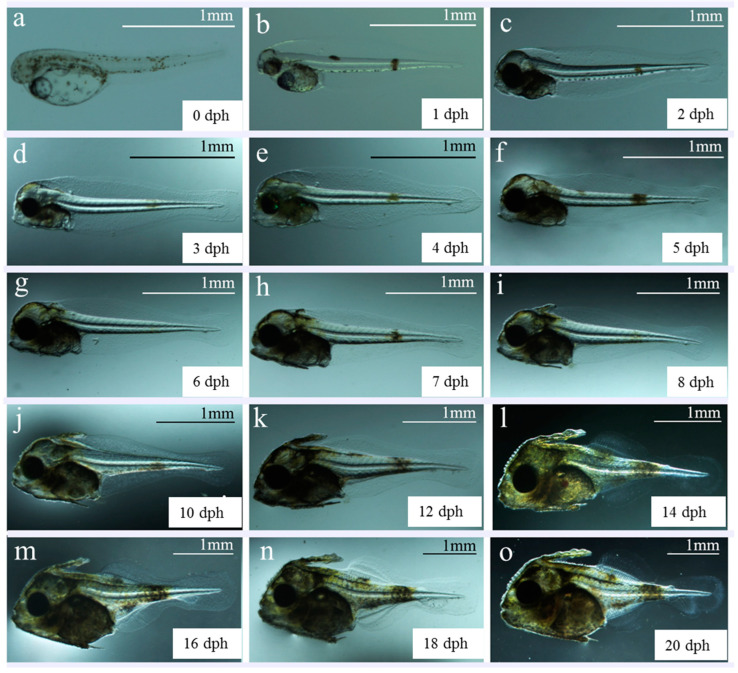

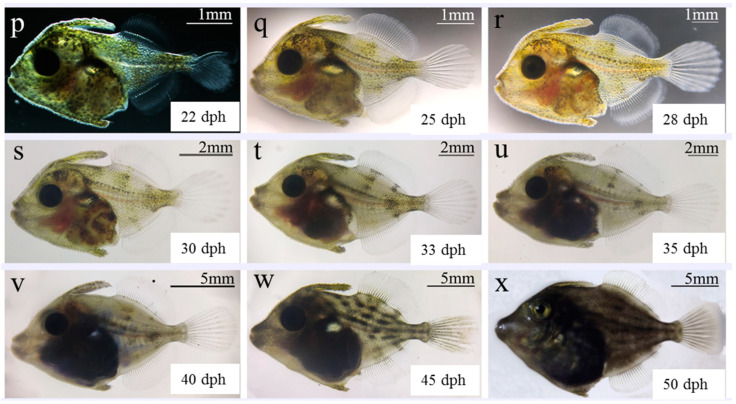
Morphologic changes in larval, juvenile, and young *S. cirrhifer*. (**a**) larval fish at 0 days post-hatching (dph); (**b**) larval fish at 1 dph; (**c**) larval fish at 2 dph; (**d**) larval fish at 3 dph; (**e**) larval fish at 4 dph; (**f**) larval fish at 5 dph; (**g**) larval fish at 6 dph; (**h**) larval fish at 7 dph; (**i**) larval fish at 8 dph; (**j**) larval fish at 10 dph; (**k**) larval fish at 12 dph; (**l**) larval fish at 14 dph; (**m**) larval fish at 16 dph; (**n**) larval fish at 18 dph; (**o**) larval fish at 20 dph; (**p**) larval fish at 22 dph; (**q**) juvenile fish at 25 dph; (**r**) juvenile fish at 28 dph; (**s**) juvenile fish at 30 dph; (**t**) juvenile fish at 33 dph; (**u**) juvenile fish at 35 dph; (**v**) juvenile fish at 40 dph; (**w**) young fish at 45 dph; (**x**) young fish at 50 dph.

The morphological characteristics of S. *cirrhifer* post-embryo from 0 to 50 dph are shown in Figure 2, and its characteristics are described in detail in Table 2. The yolk sac of newly hatched larvae was visible in the abdomen, with an average long diameter of 0.53 ± 0.03 mm and short diameter of 0.28 ± 0.03 mm. An oil globule was observed in front of the yolk sac, with an average diameter of 0.18 ± 0.01 mm. The mouth and anus were closed; the larvae were unable to swim and were suspended upside down in the water, constituting the pre-larval phase (Figure 2a–d). At 4 dph, the yolk sac was completely absorbed, and the oil globules were almost invisible. The oral fissure formed, and the digestive tract was connected, constituting the post-larval phase. Subsequently, fins appeared and gradually differentiated, gradually improving the movement and predation abilities (Figure 2e–p). After 22 dph, in the juvenile phase, fish were evenly distributed in the water and successfully swam against the current of water. Juveniles were gradually fed compound feed (Figure 2q–v). During this phase, the juveniles completed metamorphosis, with body and fin morphologies similar to those of adult fish (Figure 2w). At 50 dph, young fish exhibited strong swimming and avoidance, with an ~80% individual survival rate. Their morphological structures and ecological habits were the same as those of adult fish (Figure 2x).

### 3.3. Growth Pattern of Larvae and Juvenile

**Figure 3:**
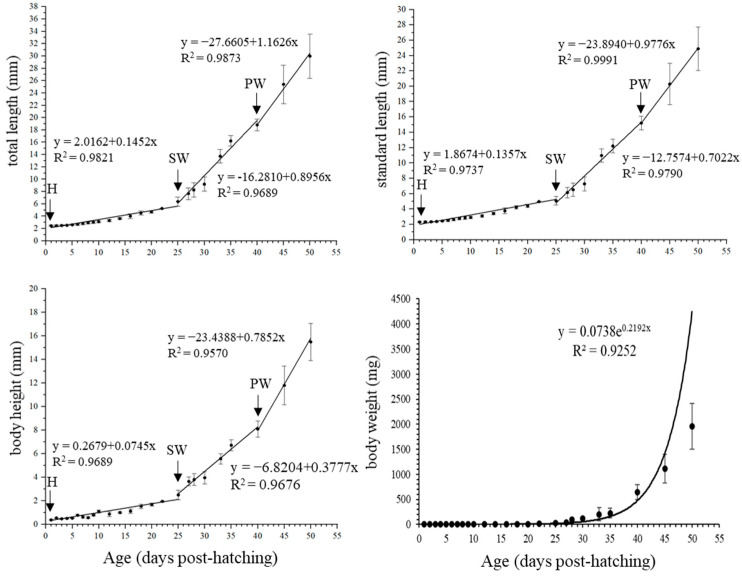
The growth patterns during early development stages of *S. cirrhifer* (H: hatching; SW: start of weaning; PW: post-weaning). The relationship was expressed by the equation Y = a + kX, where Y is the measured parameters and X is age; a is the intercept and k is the slope. Growth pattern of BW was modelled by an exponential function with the equation Y = be^kX^, where Y is BW, X is age, and k is slope. The correlation coefficient, R2, was calculated.

From 0 to 50 dph, the TL, SL, BH, and BW on each day are shown in Table S1 (Supplementary Material). From 0 to 50 dph, the TL increased from 2.45 ± 0.06 to 29.97 ± 3.61 mm, and BW increased from 0.15 ± 0.02 to 1356.86 ± 461.81 mg. As shown in Figure 3, the linear regressions of TL, SL, and BH of the larvae and juveniles were plotted against age, with two inflexion points, respectively. However, the BW showed exponential growth. All correlation coefficients (R value) were greater than 0.90. The first inflexion point of TL, SL, and BH occurred at 25 dph with TL of 6.39 ± 0.73 mm, when weaning was initiated and growth accelerated. The slopes (mean growth rates) increased from 0.1452 to 0.8956 mm day^−1^ (TL), from 0.1357 to 0.7022 mm day^−1^ (SL), and from 0.0745 to 0.3777 mm day^−1^ (BH) (Figure 3, SW). The second inflexion point occurred at 40 dph with a TL of 18.81 ± 0.97 mm, when the larvae were fed the formulated feed and growth accelerated again, and the slopes increased to 1.1626 mm day^−1^ (TL), 0.9776 mm day^−1^ (SL), and 0.7852 mm day^−1^ (BH) (Figure 3, PW).

## 4. Discussion

### 4.1. Characteristics of Fertilized Eggs

The fertilized egg of *S. cirrhifer* was a demersal egg with stickiness, which is similar to that reported in the grass pufferfish *Takifugu niphobles* [31]. However, compared to *S. cirrhifer*, the stickiness of *T. niphobles* eggs was not strong. In this study, we observed that the initial eggs of *S. cirrhifer* had strong stickiness, which decreased after hatching, similar to that of the black scraper *Thamnaconus modestus* [32]. Furthermore, the first fertilized egg of *S. cirrhifer* contained multiple oil globules, while newly hatched larvae contained only a single oil globule. It was observed that the oil globules were initially distributed in the center of the egg, and they gradually moved towards the vegetal pole during the process of cleavage. They began to fuse during muscular contraction and melted into a larger oil globule during heart pulsation. Similar phenomena were observed in other species. For example, in the fat greenling *Hexagrammos otakii* [33], multiple oil globules melted into 1–5 oil globules, while they melted into 1–3 globules in the rabbitfish *Siganus oramin* [34]. Furthermore, in the medaka *Oryzias melastigma*, the fusion of oil globules into a single globule occurred during the transition from the blastocyst to gastrula stages [35]. In the *T. modestus*, oil globules began to fuse during the tail-bud stage, and they fused into a large oil globule during the muscular contraction stage [32], which was slightly earlier than in the *S. cirrhifer* observed in this study. Therefore, the fusion period of an oil globule may be regarded as an important characteristic during the embryonic development of the *S. cirrhifer*.

In Table 3, we compare the characteristics of embryo development between *S. cirrhifer* and other filefish species. In the present study, the *S. cirrhifer* egg diameter was similar to that of *T. modestus* [32], 23.2% smaller than that of Japanese inflator filefish *Brachaluteres ulvarum* [36], and 18.9% larger than that of leatherjacket *Paramonacanthus japonicus* and White-spotted pygmy filefish *Rudariu ercodes* [37]. The embryonic incubation time for *S. cirrhifer* was 4% shorter than that of *T. modestus*, with the shortest and longest times observed in *P. japonicus* and *B. ulvarum*, respectively. Among these five species, the embryonic developmental characteristics were most similar between *S. cirrhifer* and *T. modestus*.

### 4.2. Embryonic Development

In this study, the eye primordia of *S. cirrhifer* appeared before the formation of sarcomeres, which is similar to the American shad *Alosa sapidissima* [38] and rock bream *Oplegnathus fasciatus* [39]. However, differently, the eye primordia of pufferfish *T. obscurus* appeared after the formation of sarcomeres [40]. After 15 h 10 min of fertilization during the formation of segments, the Kupffer’s vesicle appeared above the tail of the embryo with a transparent spherical cyst. After 25 h 40 min, the tail bud of the embryo became dissociated, and the Kupffer’s vesicle disappeared. Studies have shown that the early appearance of the Kupffer’s vesicle may be related to the digestion and absorption of nutrients, which may promote the absorption of yolk [41]. The cells of Kupffer’s vesicle may participate in the formation of the embryonic digestive tract during embryonic development [42]. There are also reports that the appearance of Kupffer’s vesicle initiated asymmetric development of the embryo on both sides of the symmetry axis of the embryo, creating conditions for the formation of various organs [43,44]. However, the function and mechanism of the Kupffer’s vesicle during embryonic development of *S. cirrhifer* still remain unclear in this study.

The embryonic development of fish is influenced by various factors, such as the quality of eggs and external environment [45]. Studies have shown that during the breeding season, the supply of nutrition for parent fish, especially lipid nutrition, may directly affect embryonic development [46,47,48]. Furthermore, in the fish that spawned in batches, the quality of eggs appeared different. In this study, the quality of eggs in the early and late stages of spawning was poor, and the hatching rate of eggs was also greatly lower than that during the peak period. Studies show that the embryo development of fish in the early stages is very sensitive to changes in the external environment [49]. In this study, it was observed that most fertilized eggs that were not successfully hatched showed arrested development during the stages of the cleavage and early gastrula. They displayed uneven cleavage, decreased viscosity and transparency, and the surface of the fertilized egg turning white. Studies show that that the optimal incubation temperature for silver rasbora *Rasbora argyrotaenia* eggs is 30 °C, which shows the best results of the hatching and survival rate of larvae [50]. Increased temperature is beneficial for accelerating the hatching rate of fertilized eggs in an appropriate range, but excessively high temperature may increase the malformation rate of embryos [51,52]. Further studies should focus on the effects of water temperature on the embryo development in *S. cirrhifer*.

### 4.3. Growth of Larvae and Juveniles

Weaning is a process to gradually replace live feeds with artificial diets in fish larvae [53]; therefore, this is not a natural process but rather is imposed artificially by the aquaculturist. In practice, the weaning of most temperate marine fish species such as yellowtail kingfish *Seriola lalandi* is usually commenced after metamorphosis [54]. In the present study, our feeding strategy initiated *B. plicatilis* from 3 to 25 dph, *A. salina* nauplii from 25 to 40 dph, and weaning to the formulated feed from 40 dph. In our previous study on *S. senegalensis*, the weaning period is also from 40 dph [30]. However, it suggested that the best weaning was 16–22 dph in the golden pompano *Trachinotus ovatus* [55]. Therefore, weaning presented a bottleneck in *S. cirrhifer* culture, which should be focused on in a future study.

In this study, we found two distinct inflexion points occurring in the growth curves of *S. cirrhifer* larvae, which may be related to metamorphosis, weaning, and transitions in feeding habits. Studies show that most fish have growth inflexion points in the early stages such as the grey mullet *Chelon labrosus* [56], as well as our previous studies on *S. cirrhifer* and *T. modestus* [57]. Furthermore, previous studies have shown that the phenomenon of inflexion points in the early stages of fish is related to their survival strategies and developmental mechanisms during long-term evolution, aiming to ensure that the most important organs for early survival are prioritized for development. For example, in the chum salmon *Oncorhynchus keta* [58] and *C. labrosus* [56], they prioritize the development of sensory, feeding, respiratory, and motor organs.

In this study, the inflexion points of total length, standard length, and body height appeared at 25 dph and 40 dph, respectively, with an accelerated growth in these stages. At 25 dph, with the development of the fin as the main swimming organ, the fish developed from the larval to juvenile stages, showing a dietary transition from *B. plicatilis* to *A. salina* nauplii, and the growth accelerated. This phenomenon was also observed in the larval rearing of *T. modestus* [32], rockfish *Sebastes schlegeli* [59], and yellow catfish *Pelteobagrus fulvidraco* [60]. At 40 dph, the main digestive organs of juveniles had developed well and were able to feed on formulated feed. The food intake increased sharply, and the second growth acceleration period occurred. Therefore, it is suggested that the bait and changes in feeding habits have a significant impact on the growth rate of larval fish in *S. cirrhifer*.

## 5. Conclusions

We derived the following results: (1) *S. cirrhifer* had spherical, sticky, and transparent fertilized eggs with multiple oil globules, and oil globules fused at the stage of muscular contraction and melted into a larger oil globule during heart pulsation; (2) the embryonic development of *S. cirrhifer* was divided into eight phases from pre-cleavage to hatching, lasting 48 h, and the overall process is similar to that of *T. modestus*; (3) the weaning period occurred from 40 dph with feeding changing from live feeds to artificial diets; and (4) two inflexion points occurring in the growth curves of larvae and juveniles were found to be associated with metamorphosis and transitions in feeding habits.

As the global output and quality of *S. cirrhifer* gradually decrease, these results may provide guidance to improve artificial propagation and early life stage success in filefish species. Further studies are essential for understanding the mechanisms of growth and reproductive regulation from hatching to maturation in this species.

## Figures and Tables

**Table 1:** Embryonic characteristics and duration of development in *S. cirrhifer*.

Developmental Stage	Developmental Time (h:min)	Description	Figure Panel
Fertilized egg	0	Eggs spherical in shape; yolk is transparent with multiple oil globules and ooplasm is evenly distributed	Figure 1A
Blastodisc formation	0:30	Cytoplasm migrates toward the animal pole to form blastodisc.	Figure 1B
2-cell	1:10	First cleavage: blastodisc divides via meridional cleavage to form two equal cells	Figure 1C
4-cell	1:20	Second cleavage: 2 × 2 blastomere shape	Figure 1D
8-cell	1:50	Third cleavage: 2 × 4 array of blastomere	Figure 1E
16-cell	2:10	Fourth cleavage:4 × 4 blastomere shape	Figure 1F
32-cell	2:30	Fifth cleavage: 4 × 8 blastomere shape	Figure 1G
64-cell	2:55	Sixth cleavage: 64 blastomeres ranked irregularly	Figure 1H
Multi-cell	3:25	Variable blastomere size and shape; cleavage occured asynchronously.	Figure 1I
Morula	4:30	Multilayer cells were formed at animal pole.	Figure 1J
Early blastula	5:20	Blastoderm composed of many blastomeres with unclear border, and blastoderm hunch was high.	Figure 1K
Late blastula	6:20	Epibolic cells increased in number and blastoderm hunch gradually lowered.	Figure 1L
Early gastrula	7:20	Germ ring appeared and blastoderm epiboled toward vegetal pole to 1/3 of yolk sac.	Figure 1M
Middle gastrula	9:40	Germ ring was distinct and blastoderm epiboled 1/2 of the yolk sac; embryonic shield is formed.	Figure 1N
Late gastrula	11:40	Blastoderm epiboled 3/4 of the yolk sac; embryonic shield elongated with the involution of blastoderm cells.	Figure 1O
Neurula	14:00	Yolk plug was formed; embryonic body and neural plate was formed; optic vesicles were formed.	Figure 1P
Formation of somite	15:10	Kupffer’s vesicle was visible; somite begins to form	Figure 1Q
Formation of optic vesicle	20:40	Optic vesicles were formed.	Figure 1R
Formation of tail bud	25:40	Tail dissociated from yolk sac to form caudal bud; Kupffer’s vesicle disappeared.	Figure 1S
Formation of auditory capsule	28:55	Auditory capsules were formed bilaterally on the hindbrain.	Figure 1T
Muscular contraction	32:40	muscles contracted and tail wobbled intermittently; punctate yellow-green pigment appeared.	Figure 1U
Heart pulsation	37:40	Heart started to beat.	Figure 1V
Hatching	48:00	Embryonic body surrounded yolk sac in one lap and twisted frequently; head and tail wobbled dramatically.	Figure 1W

**Table 2:** Characteristics of larval, juvenile, and young *S. cirrhifer*.

Phase	dph	Morphological Characteristics	Ecological Habits
Pre-larva a	0	Larvae have an oil globule in front of the yolk sac. The optic capsule and crystal are colorless and transparent. The digestive tract is attached to the upper portion of the yolk sac without opening. The anus is closed. The dorsal and gluteal fins are connected with the caudal fin membrane, without pigment distribution. There are scattered yellow-green spots on the back, abdomen, and back of the body segment.	The larvae hang upside down in the pool and are evenly distributed.
b	1	Yolk sac and oil globule are clearly reduced, and skull top is lifted upward. A small pigment appears in the optic capsule and crystal, the back and abdomen of the front end of the segment, and the rear end of the segment gathers to form three massive yellow-green spots. The pectoral fin membrane is inverted as triangular, and the digestive tract is thickened.	
c–d	3	Yolk sac disappears and the oil globule is almost invisible. The eye develops rapidly, the optic capsule and crystal are black, and the mouth fissure is initially formed. The first bend of the digestive tract occurs. The anus is formed, and digestive tract runs through. The color patches on the back and abdomen in the front body segment disappear.	Larval cluster distributed in the upper layer of the water, and some larvae feed on S-rotifers.
Post- larva e–f	4	Oil globule disappears, and the color of visceral mass deepens. The digestive tract is filled with food, and a little star-shaped pigment appears on the top of the head. The dorsal fin spine primordia appears, and the swim bladder appears and inflates. The oral fissure increases and expands, and the jaw fully opens.	
g–i	6–8	The upper and lower jaws of larvae are gradually covered by leathery skin, and the head-to-body proportion increases. The dorsal fin spines, abdominal girdle bones, and abdominal fin spines are formed, with dense distribution of melanin and yellow pigment in the head. The yellow-green spots on the body segments disappear. Three reddish-brown patches gradually grow and spread throughout the body.	Most larvae feed on L-rotifer with weak phototaxis.
j–k	10–12	Pigmentation increases in the upper and lower jaw and trunk. The dorsal, anal, and caudal fin arms thicken.	
l–m	14–16	Head appears with protruding scales. The first dorsal fin spine shows inverted spines, and the waistband bone and abdominal fin spine move downward, resulting in a substantial increase in body height. Fin differentiation occurs in the dorsal, anal, and pectoral fins, with widespread distribution of yellow and melanin on the body surface. Swim bladder is clear.	Swimming ability enhanced, resulting in obvious clustering phenomenon.
m–o	16–20	The waistband bone thickens and the abdominal fin spines atrophy. The lower lobe of the caudal fin begins to protrude, and radial elastic filaments of the caudal fin appear. The caudal vertebrae are flat.	
o–p	20–22	The spine of the abdominal fin falls off, and the girdle bone continues to thicken and differentiate into thorns. The dorsal and anal fins exhibit obvious growth, and fins begin to take shape. The lower lobe of caudal fin protrudes backward and the end of the notochord tilts upwards. Protruding scales appear on the body surface.	Larvae feeds vigorously and consume considerable amounts of *Artemia* nauplii.
p–q	22–25	The end of the notochord continues to tilt upwards, forming a tail fan. The trunk is evenly distributed with protruding circular punctate scales. The snout is covered with leathery epidermis, and the mouth begins to round.	
Juvenile q–r	25–28	Each fin is mostly formed, with a small amount of primitive fin membrane remaining at the end of the dorsal and anal fins. The caudal fins are segmented and not branched.	Juveniles are uniformly distributed in the water.
r–s	28–30	The scales of the trunk develop sharp thorns.	
t–u	33–35	The snout extends, the caudal fins begin to form branches, and the original membranes of each fin disappear.	
u–v	35–40	There are 12 tail fin strips, consistent with adult fish, and the ends continue to branch.	Juveniles begins to consume compound feed, and often kill each other.
v–w	40–45	Melanin increases, with horizontal dark stripes appearing on the trunk. Pigmented spots appear on the tail fins. The counts of dorsal fins and anal fins are 34–36 and 32–34, respectively. The body shape of juveniles and morphology of each fin are similar to those of adult fish.	Metamorphosis is complete, with obvious the size differentiation of juveniles.
Young w–x	50	The brown dark lines on the trunk are more prominent; the pigmented spots on the dorsal and anal fins are deepened. The tail fins are densely yellow. The fish are covered with scales.	Fish have strong swimming and avoidance abilities, and have similar ecological habits as adult fish.

**Table 3:** Comparison of embryonic development characteristics among five Monacanthidae fish.

Species	Egg Diameter (mm)	Number of Oil Globules	Diameter of Oil Globules (μm)	Incubation Temperature (°C)	Sum of Temperature (°C · h)	Hatching Time	Reference
*Thamnaconus* *modestus*	0.59–0.63	18–83	35–206	20.5–21.5	1050	50 h	[32]
*Brachaluteres* *ulvarum*	0.82	20	30–130	19–22	3225–3600	150 h	[36]
*Paramonacanthus japonicus*	0.53	10–20	30–70	29.0–29.3	841–850	29 h	[37]
*Rudarius* *ercodes*	0.53	1–3	120–170	20.7–21.3	1283–1311	62 h 39 min	[37]
*Stephanolepis* *cirrhifer*	0.61–0.63	4–6	100–142	23.6	1132.8	48 h	This study

## Data Availability

The data presented in this study are available on request from the corresponding author due to ethical, legal, or privacy restrictions.

## Supplementary Materials

The following supporting information can be downloaded at: https://www.mdpi.com/article/10.3390/ani15081124/s1, Table S1: The growth indexes of *Stephanolepis cirrhifer*.

## References

[1] Nelson J.S., Grande T.C., Wilson M.V. (2016). Fishes of the World.

[2] Alien G.R., Amaoka K., Anderson W.D., Bellwood D.R., Bohlke E.B., Bradbury M.G., Carpenter K.E., Caruso J.H., Cohen A.C., Cohen D.M. (2000). A checklist of the fishes of the South China Sea. Raffles Bull. Zool..

[3] Nakabo T. (2022). Fishes of Japan: With Pictorial Keys to the Species.

[4] Miyajima-Taga Y., Masuda R., Yamashita Y. (2016). Larvae of the threadsail filefish *Stephanolepis cirrhifer* feed on eggs and planulae of the jellyfish Aurelia sp. under laboratory conditions. Plankton Benthos Res..

[5] Youn C.H. (2002). Fishes of Korea with Pictorial Key and Systematic List.

[6] Kim P.D. (2007). Miniature Guide for Whole Korean Fish Seoul.

[7] Minami T., Kanemaru M., Iwata K., Kuwahara M., Amano K., Mizuta A., Maeda N., Nishiki I., Tue Y., Yoshida T. (2013). Pathogenicity of Streptococcus iniae and Lactococcus garvieae in farmed thread-sail filefish and efficacy of the formalin-killed vaccines against these bacteria. Fish Pathol..

[8] Mizuno K., Miura C., Miura T. (2014). Relationships between Temperature and Growth of Thread-sail Filefish *Stephanolepis cirrhifer* and Black Scraper *Thamnaconus modestus*. Aquacult. Sci..

[9] Yamada T., Sugihara Y., Takami I., Suga K., Kanai K. (2013). Protective efficacy of a commercial β-hemolytic Streptococcus vaccine for Japanese flounder against Streptococcus iniae infection of threadsail filefish. Fish Pathol..

[10] Park S.Y., Lee N.Y., Kim S.H., Cho J.I., Lee H.J., Ha S.D. (2014). Effect of ultraviolet radiation on the reduction of major food spoilage molds and sensory quality of the surface of dried filefish (*Stephanolepis cirrhifer*) fillets. Food Res. Int..

[11] Miyajima Y., Masuda R., Kurihara A., Kamata R., Yamashita Y., Takeuchi T. (2011). Juveniles of threadsail filefish, *Stephanolepis cirrhifer*, can survive and grow by feeding on moon jellyfish *Aurelia aurita*. Fish. Sci..

[12] Garibaldi L., Caddy J.F. (2004). Depleted Marine Resources: An Approach to Quantification Based on the FAO Capture Database.

[13] An C.M., An H.S., Lee J.W., Hong S.W. (2013). New polymorphic microsatellite loci of threadsail filefish, *Stephanolepis cirrhifer* (Teleostei, Monacanthidae), from Korean waters. Genet. Mol. Res..

[14] Matsuura K., Motomura H., Khan M. (2019). *Stephanolepis cirrhifer*. IUCN Red List. Threat. Species.

[15] Markevich A.I., Balanov A.A. (2011). Finding of threadsail filefish *Stephanolepis cirrhifer* (Temminck et Schlegel, 1850) rare for Peter the Great Bay, Sea of Japan. J. Ichthyol..

[16] Allen J.J., Akkaynak D., Sugden A.U., Hanlon R.T. (2015). Adaptive body patterning, three-dimensional skin morphology, and camouflage measures of the slender filefish *Monacanthus tuckeri* on a Caribbean coral reef. Biol. J. Linn. Soc..

[17] An H.S., Hong S.W., Kim E.M., Myeong J.I. (2011). Comparative genetic diversity of wild and hachery populations of Korean threadsail filefish *Stephanolepis cirrhifer* using cross-species microsatellite markers. Genes Genom..

[18] Yoon M., Park W., Nam Y.K., Kim D.S. (2012). Shallow population genetic structures of Thread-sail Filefish (*Stephanolepis cirrhifer*) populations from Korean coastal waters. Asian Australas. J. Anim. Sci..

[19] Zhang J., Chen P.M., Chen G.B., Fang L.C., Tang Y. (2014). Acoustic target strength measurement of banded grouper (*Epinephelus awoara* (Temming and Schlegel, 1842)) and threadsial filefish (*Stephanolepis cirrhifer* (Temming & Schlegel, 1850)) in the South China Sea. J. Appl. Ichthyol..

[20] Hossain M.A., Furuichi M. (2008). Necessity of dietary calcium supplement in file fish (*Monacanthus cirrhifer*). Bangladesh J. Fish. Res..

[21] Khosravi S., Lee S.M. (2017). Optimum dietary protein and lipid levels in juvenile filefish, *Stephanolepis cirrhifer*, feed. J. World Aquacult. Soc..

[22] Narita A., Kashiwagura M., Saito H., Okada Y., Akiyama N. (2011). Effect of different rearing conditions on larval feeding activity, food consumption, survival and growth of filefish *Stephanolepis cirrhifer* larvae. Aquacult. Sci..

[23] An H.S., Lee J.W., Hong S.W., Myeong J.I., An C.M. (2013). Population genetic structure of the Korean threadsail filefish (*Stephanolepis cirrhifer*) based on microsatellite marker analysis. Biochem. Syst. Ecol..

[24] Xu W., Zeng J., Mei W., Jiang L., Manabe S., Wu Y., Liu L. (2023). Changes in Growth and Feeding Characteristics during Early Ontogenesis in Threadsail Filefish, *Stephanolepis cirrhifer*. Animals.

[25] Boehlert G.W., Yamada J. (1991). Rockfishes of the genus Sebastes: Their reproduction and early life history. Developments in Environmental Biology of Fishes.

[26] Du R., Wang Y., Jiang H., Liu L., Wang M., Li T., Zhang S. (2010). Embryonic and larval development in barfin flounder *Verasper moseri* (Jordan and Gilbert). Chin. J. Oceanol. Limnol..

[27] Løkkeborg S., Siikavuopio S.I., Humborstad O.B., Utne-Palm A.C., Ferter K. (2014). Towards more efficient longline fisheries: Fish feeding behaviour, bait characteristics and development of alternative baits. Rev. Fish Biol. Fisher..

[28] Song Y.Q., Cheng F., Zhao S.S., Xie S.G. (2019). Ontogenetic development and otolith microstructure in the larval and juvenile stages of mandarin fish *Siniperca chuatsi*. Ichthyol. Res..

[29] Van Snik G.M.J., Van den Boogaart J.G.M., Osse W.M. (1997). Larval growth patterns in *Cyprinus carpio* and *Clarias gariepinus* with attention to the finfold. J. Fish Biol..

[30] Liu L.M., Zeng J., Wang J.L., Liu Y., Mei W.P., Wu Y.Q., Wang C.W., Xu W.G. (2024). Early ontogenesis from embryo to juvenile in Senegalese sole *Solea senegalensis* under laboratory conditions. J. Fish Biol..

[31] Gallego V., Yoshida M., Kurokawa D., Asturiano J.F., Fraser G.J. (2017). Embryonic development of the grass pufferfish (*Takifugu niphobles*): From egg to larvae. Theriogenology.

[32] Guan J., Ma Z., Zheng Y., Guan S., Li C., Liu H. (2013). Breeding and larval rearing of bluefin leatherjacket, *Thamnaconus modestus* (Gunther, 1877) under commercial scales. Int. J. Aquac..

[33] Hu F.W., Pan L., Gao F.X., Jian Y.X., Zhang S.C., Wang X., Guo W. (2012). Embryonic development of *Hexagrammos otakii* and its relationship with incubation temperature. Prog. Fish. Sci..

[34] Huang X.L., Yang Y.K., Li T., Huang Z., Yu W., Lin H.Z. (2018). Morphology and growth of larval, juvenile and young *Siganus oramin*. South China Fish. Sci..

[35] Wang Y.H., Liu H.J., Yu D.D., Li Y.Q., Guan S.G., Liu Y. (2017). Observation of embryonic development of marine medaka *Oryzias melastigma*. Mar. Sci..

[36] Akagawa I., Tsukamoto Y., Okiyama M. (1995). Sexual dimorphism and pair spawning into a sponge by the filefish, *Brachaluteres ulvarum*, with a description of the eggs and larvae. Jap. J. Ichthyol..

[37] Kawase H., Nakazono A. (1994). Embryonic and pre-larval development and otolith increments in two filefishes, *Rudarius ercodes* and *Paramonacanthus japonicus* (Monacanthidae). Jap. J. Ichthyol..

[38] Gao X.Q., Liu Z.F., Huang B., Wang Y.H., Xue G.P., Qin W.L., Guan C.T., Hong L. (2017). Morphological and histological observation of the embryo of American Shad (*Alosa sapidissima*). Prog. Fish. Sci..

[39] He T., Xiao Z.Z., Liu Q.H., Ma D.Y., Xu S.H., Xiao Y.S., Li J. (2012). Histological observation of eye ontogeny in rock bream larvae (*Oplegnathus fasciatus*). Mar. Sci..

[40] Mo G.Y., Hu G.D., Zhou Y.F. (2009). Observation on the embryonic development of *Takifugu obscurus*. Freshw. Fish..

[41] Brummett A.R., Dumont J.N. (1978). Kupffer’s vesicle in *Fundulus heteroclitus*: A scanning and transmission electron microscope study. Tissue Cell.

[42] Rosenthal H., Fond M. (1973). Biological observations during rearing experiments with the garfish *Belone belone*. Mar. Biol..

[43] Essner J.J., Amack J.D., Nyholm M.K., Harris E.B., Yost H.J. (2005). Kupffer’s vesicle is a ciliated organ of asymmetry in the zebrafish embryo that initiates left-right development of the brain, heart and gut. Development.

[44] Okabe N., Burdine R.D. (2008). Fluid dynamics in zebrafish Kupffer’s vesicle. Dev. Dynam..

[45] Humphrey C., Weber M., Lott C., Cooper T., Fabricius K.J.C.R. (2008). Effects of suspended sediments, dissolved inorganic nutrients and salinity on fertilisation and embryo development in the coral *Acropora millepora* (Ehrenberg, 1834). Coral Reefs.

[46] Duray M., Kohno H., Pascual F. (1994). The effect of lipid-enriched broodstock diets on spawning and on egg and larval quality of hatchery-bred rabbitfish *Siganus guttatus*. Philipp. Sci..

[47] Furuita H., Tanaka H., Yamamoto T., Shiraishi M., Takeuchi T. (2000). Effects of n-3 HUFA levels in broodstock diet on the reproductive performance and egg and larval quality of the Japanese flounder, *Parolichthys olivaceus*. Aquaculture.

[48] Li Y.Y., Chen W.Z., Sun Z.W., Chen J.H., Wu K.G. (2005). Effects of n-3HUFA content in broodstock diet on spawning performance and fatty acid composition of eggs and larvae in *Plectorhynchus cinctus*. Aquaculture.

[49] Jobling M. (2012). Fish in aquaculture environments. Aquaculture and Behavior.

[50] Syafariyah N.K., Sulmartiwi L., Budi D.S. (2023). Incubation temperature effects on some hatching parameters of silver rasbora (*Rasbora argyrotaenia*) egg. J. Appl. Aquacult..

[51] Martell D.J., Kieffer J.D., Trippel E.A. (2005). Effects of temperature during early life history on embryonic and larval development and growth in haddock. J. Fish Biol..

[52] Kupren K., Mamcarz A., Kucharczyk D., Prusińska M., Krejszeff S. (2008). Influence of water temperature on eggs incubation time and embryonic development of fish from genus Leuciscus. Pol. J. Nat. Sci..

[53] Saputra A., Suryaningrum L.H., Sunarno M.T.D., Samsudin R., Kholidin E.B., Prihadi T.H., Widyastuti Y.R., Murniasih S., Kontara E.K.M., Taukhid T. (2024). Enhancing early weaning strategies through artificial feeding regimes for *Channa striata* larvae. Egypt. J. Aquat. Res..

[54] Ma Z.H., Qin J.G., Hutchinson W., Chen B.N., Song L. (2014). Responses of digestive enzymes and body lipids to weaning times in yellowtail kingfish *Seriola lalandi* (Valenciennes, 1833) larvae. Aquac. Res..

[55] Ma Z., Zheng P., Guo H., Zhang N., Wang L., Jiang S., Qin J.G., Zhang D. (2015). Effect of weaning time on the performance of *Trachinotus ovatus* (Linnaeus 1758) larvae. Aquacult. Nutr..

[56] Khemis I.B., Gisbert E., Alcaraz C., Zouiten D., Besbes R., Zouiten A., Masmoudi A.S., Cahu C. (2013). Allometric growth patterns and development in larvae and juveniles of thick-lipped grey mullet *Chelon labrosus* reared in mesocosm conditions. Aquac. Res..

[57] Liu L.M., Zeng J., Zhang Z., Wang J.L., Mei W.P., Wang C.W., Liu Z.P., Xu W.G. (2024). Changes in growth, morphology, and levels of digestive enzymes and growth-related hormones in early ontogeny of black scraper, *Thamnaconus modestus*. Front. Mar. Sci..

[58] Song H.J., Liu W., Wang J.L., Tang F.J. (2013). Allometric growth during yolk-sac larvae of chum salmon (*Oncorhynchus keta* Walbaum) and consequent ecological significance. Acta Hydrobiol. Sin..

[59] Wang R.L., Wang Z.B., Jiang H.B., Du R.B., Liu L.M. (2018). Growth and feeding characteristics of *Sebastes schlegeli* in specific developmental periods. J. Ocean Univ. China.

[60] Huang Q., Jiang S., Qi K. (2012). The changes in growth, survival, food intake and body composition of *Pseudogrus fulvidraco* larvae and juveniles fed with *Artemia nauplii*. J. Fish. Sci. China.

