# Supplementary material for: Ontogenesis from Embryo to Juvenile in Threadsail Filefish, Stephanolepis cirrhifer
Source: Animals (Basel). 2025 Apr 13;15(8):1124. doi: 10.3390/ani15081124 (PMC12024050; doi:10.3390/ani15081124)
Supplement: Supplementary file 1 [file animals-15-01124-s001.zip › animals-3553680-supplementary.pdf]

Table S1. The growth indexes of *Stephanolepis cirrhifer*

Age (dph)	Total length (mm)	Standard length (mm)	Body height (mm)	Body weight (mg)
1	2.45±0.06 ^{ab}	2.30±0.07 ^{a}	0.47±0.04 ^{a}	0.15±0.02 ^{a}
5	2.60±0.07 ^{ab}	2.44±0.06 ^{ab}	0.56±0.03 ^{a}	0.28±0.02 ^{a}
7	2.82±0.19 ^{ab}	2.64±0.18 ^{abc}	0.64±0.08 ^{a}	0.35±0.10^{a}
10	3.11±0.17 ^{ab}	2.90±0.16 ^{abc}	0.81±0.12 ^{ab}	0.45±0.09 ^{a}
14	3.62±0.15 ^{abc}	3.41±0.16 ^{abc}	1.02±0.07 ^{abc}	0.62±0.11 ^{a}
18	4.53±0.30 ^{abc}	4.22±0.21 ^{abcd}	1.46±0.19 ^{bc}	1.37±0.51 ^{a}
22	5.26±0.16 ^{bc}	4.96±0.05 ^{bcde}	1.94±0.02 ^{cd}	11.12±1.04 ^{ab}
24	6.39±0.73 ^{cd}	5.07±0.56 ^{bcde}	2.65±0.41 ^{de}	41.10±9.89 ^{b}
28	8.27±0.95 ^{d}	6.51±0.68 ^{de}	3.49±0.38 ^{ef}	90.47±36.48 ^{bc}
30	9.20±1.16 ^{d}	7.27±0.90 ^{e}	3.75±0.51^{f}	113.33±15.80 ^{cd}
32	13.73±1.08 ^{e}	10.96±0.88 ^{f}	5.46±0.44 ^{g}	150.56±71.21 ^{d}
35	16.21±1.14 ^{ef}	12.21±0.84 ^{fg}	6.72±0.17 ^{h}	225.67±97.10 ^{e}
40	18.81±0.97 ^{f}	15.20±0.90 ^{g}	8.24±0.69 ^{i}	644.21±151.11 ^{f}
45	25.40±3.11 ^{g}	20.28±2.70 ^{h}	11.50±1.65 ^{j}	1014.42±286.08 ^{g}
50	29.97±3.61 ^{h}	24.89±2.83 ^{i}	13.71±1.57 ^{k}	1356.86±461.81 ^{h}
